# Corrosion Monitoring by Plastic Optic Fiber Sensor Using Bi-Directional Light Transmission

**DOI:** 10.3390/s24103229

**Published:** 2024-05-19

**Authors:** Liang Hou, Shinichi Akutagawa

**Affiliations:** Department of Civil Engineering, Kobe University, 1-1, Rokkodai-cho, Nada-ku, Kobe 657-8501, Japan; cadax@kobe-u.ac.jp

**Keywords:** corrosion detection, plastic optical fiber sensor, dummy sensor

## Abstract

In this paper, a new sensor is proposed to efficiently gather crucial information on corrosion phenomena and their progression within steel components. Fabricated with plastic optical fibers (POF), the sensor can detect corrosion-induced physical changes in the appearance of monitoring points within the steel material. Additionally, the new sensor incorporates an innovative structure that efficiently utilizes bi-directional optical transmission in the POF, simplifying the installation procedure and reducing the total cost of the POF cables by as much as 50% when monitoring multiple points. Furthermore, an extremely compact dummy sensor with the length of 5 mm and a diameter of 2.2 mm for corrosion-depth detection was introduced, and its functionality was validated through experiments. This paper outlines the concept and fundamental structure of the proposed sensor; analyzes the results of various experiments; and discusses its effectiveness, prospects, and economic advantages.

## 1. Introduction

Monitoring the health of engineering infrastructures has become increasingly crucial, with steel corrosion emerging as one of the most prevalent structural defects requiring attention in structural health evaluations. For example, corrosion of metallic pipelines is a significant issue, leading to an exponential increase in the failure rate of water distribution networks [1,2]. Despite the fact that corrosion protection technologies have become more sophisticated, there is no consensus on which method is most effective under specific environmental conditions [3]. The structural integrity and service lives of various structures across industries must be ensured through corrosion monitoring at an acceptable cost.

Previous studies have delved into the corrosion process and monitoring technologies across various fields, aiming to provide a comprehensive overview [4,5]. These studies sought to assess corrosion initiation and progression by monitoring key factors influenced by corrosion onset, including physical appearance [6], alterations in electrochemical signals [7,8,9,10,11], specimen weight variations [12], structural deformation [13,14,15], alterations in wave propagation characteristics [16,17,18,19], and modifications in the biological corrosion environment [20]. Despite the accuracy of these corrosion monitoring methods, challenges persist, including pinpointing precise corrosion locations, detecting early-stage corrosion, determining corrosion depths, the dependence of data on monitoring environments, and cost-related challenges associated with required monitoring equipment.

To meet these challenges, a novel approach utilizing a plastic optical fiber (POF) sensor is proposed, aimed at enabling the creation of a cost-effective sensor package and an efficient data logging system for corrosion monitoring. This choice is motivated by several factors: (1) POF sensors can be manufactured at a relatively low cost; (2) the manufacturing process for these sensors is straightforward; (3) optical data captured by multiple POF sensors can be processed using graphical application software on a mobile phone; and (4) optical data or the corrosion condition can be visually confirmed if necessary. The initial experiment employing POF sensors to detect expanding corrosion zones in steel plates was conducted successfully [21], validating the factors listed above. However, the proposed method required two or more POF cables for corrosion detection: one for transmitting light to a monitoring point and another for retrieving reflected light to capture the corrosion phenomenon.

This paper introduces an improved method aimed at reducing the overall cost of POF-based corrosion detection. A novel POF sensor was developed, incorporating bi-directional light transmission in a single POF cable connected to two short POF cables with half-sized diameters, enabling the total use of POF cables by as much as 50%. Furthermore, an extremely compact dummy sensor with a length of 5 mm and a diameter of 2.2 mm for corrosion-depth detection was developed and tested. The structural details of these new sensors, functional verification, corrosion tests on steel plates, and compact dummy units are presented to demonstrate its efficiency and limitations, as well as a substantial reduction in the overall cost of corrosion monitoring.

## 2. Problem Setting for New POF Sensor

### 2.1. Scenario for Corrosion of Steel Plate

**Figure 1:**
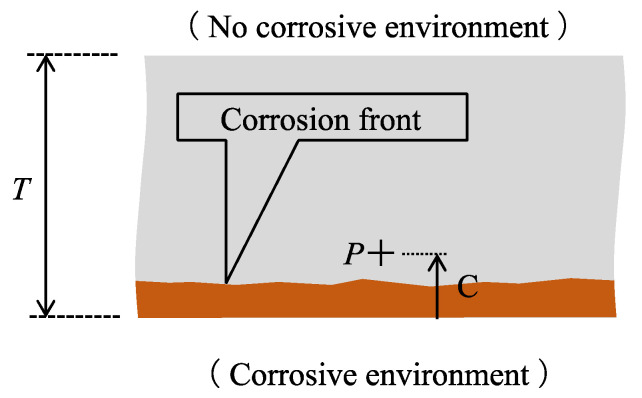
Scenario exhibiting corrosion process in a steel plate.

First, let us clarify the assumptions and fundamental strategy for monitoring steel plate corrosion using a new POF sensor based on bi-directional light transmission. Initially, we postulate that corrosion initiates on the lower surface of a steel plate with thickness *T*, as depicted in Figure 1, within a corrosive environment. Subsequently, the corrosion zone progresses upward, and the corrosion front, which may not be entirely flat, extends to a designated point *P* located at a distance *C* from the lower edge. This point is detected by a POF sensor.

### 2.2. Fundamental Principle of New POF Sensor

**Figure 2:**
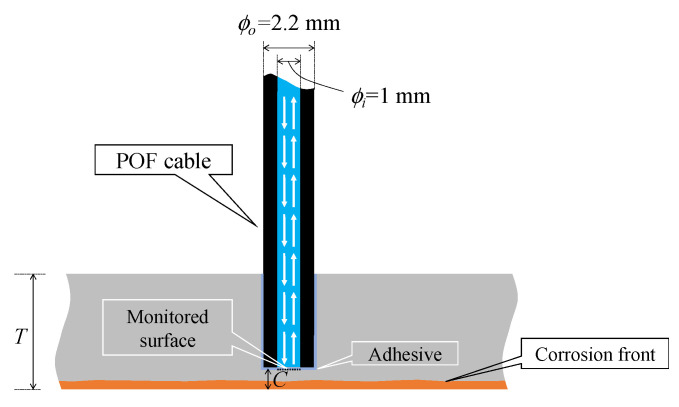
Image of new POF sensor using bi-directional transmission of light.

In order to determine whether a corrosion area has reached an arbitrary point inside a steel material, it is generally necessary to use two POFs. Firstly, the first POF is required to supply light to that point, and another POF is essential for capturing reflected light to read the arrival of the corrosion area. Additionally, to install this type of POF sensor, it is necessary to prepare installation holes large enough to accommodate at least two POFs. However, in order to reduce the total cost required for corrosion monitoring, streamline the tasks necessary for sensor installation, and minimize the impact on structures, further innovative approaches are necessary. To meet this need and facilitate corrosion detection with a minimal number of POF cables and streamline installation, it would be ideal for a new POF sensor to transmit and receive light using only one POF cable at the corrosion detection point, as illustrated in Figure 2.

**Figure 3:**
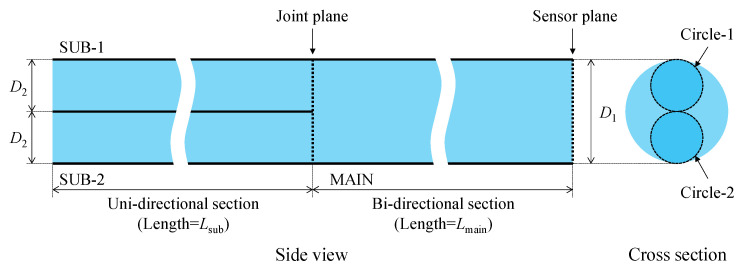
Structure of new POF sensor.

Figure 3 shows the structure of the new POF sensor specifically designed for this purpose. The MAIN fiber, with a length of *L*_main_ and a diameter of *D*_1_ (1 mm), had its right end serving as the sensor plane. Two POFs, with lengths *L*_sub_ and diameters *D*_2_ (0.5 mm), were attached to the MAIN fiber using adhesive. One of these fibers (SUB-1 fiber) was linked to a light source, while the other (SUB-2 fiber) was connected to an image-processing unit.

**Figure 4:**
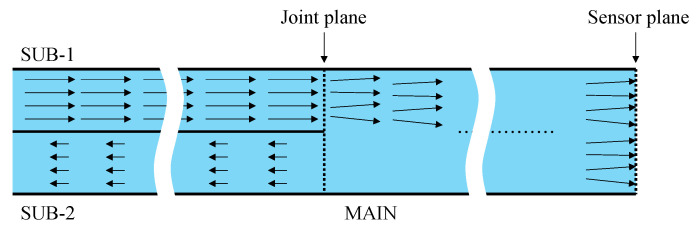
Light flow from light source to sensor plane, including scattered light by joint plane moving leftward in SUB-2.

Initially, light emitted from the light source traveled rightward through the SUB-1 fiber and reached the joint plane (Circle-1 in Figure 3) connected to the MAIN fiber. Due to the thin adhesive layer on the joint plane, some light was reflected onto the SUB-1 fiber, which was disregarded and did not affect the measurement. The majority of the light reaching the joint plane traversed the adhesive layer and continued rightward within the MAIN fiber. Although the primary direction of light was rightward, the joint plane, filled with a thin layer of adhesive, also scattered some light, causing a portion to travel leftward into SUB-2. Ultimately, light exclusively passing through Circle-1 at the left end of the MAIN fiber moved rightward, utilizing the entire cross-section of the MAIN fiber, and reached the end face, which served as the sensor plane, as shown in Figure 4.

A layer of material *A*, a thin, transparent adhesive, was placed in front of the sensor plane. On the right side of the adhesive was the material *S*, which was steel. In this configuration, light reached all positions on the sensor plane at the right end of the MAIN fiber.

**Figure 5:**
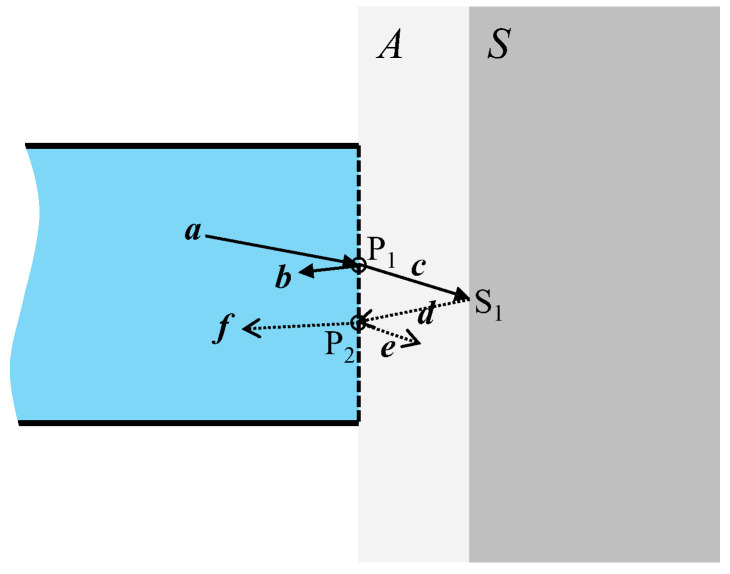
Light movement at tip of sensor. Note that the thickness of material *A* is exaggerated for the sake of visual explanation.

**Figure 6:**
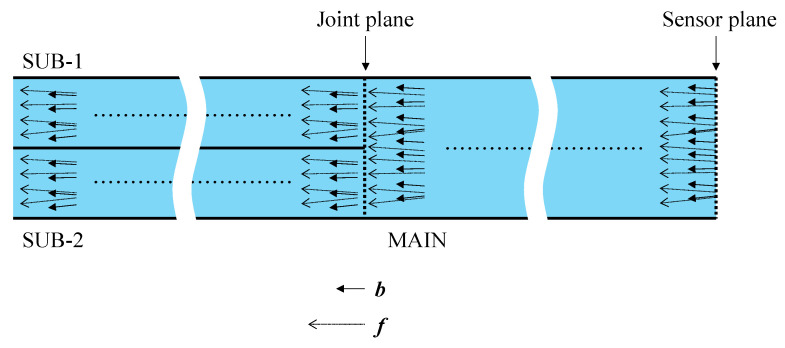
Various light paths returning from sensor plane.

In Figure 5, ***a*** represents the incident light reaching P_1_ on the sensor plane. A portion of ***a*** is reflected at the boundary between the sensor plane and material *A*, forming light ***b***, which maintains a consistent intensity and is unaffected by corrosion on the steel surface. The refracted light passing through the boundary surface traveled through material *A* as ***c*** and reached S_1_ on the surface of material *S*. The reflected light at S_1_, depicted as ***d***, was influenced by the surface condition of material *S* and arrived at P_2_ on the sensor plane. Segment ***d*** is reflected as ***e*** at the boundary between material *A* and the POF. However, the remaining refracted portion entered the POF as ***f*** and propagated leftward within the POF. This process occurred uniformly across the sensor plane, contributing to the leftward-traveling light in the new sensor, as illustrated in Figure 6.

The combined light of ***b*** (representing the light that has not reached the steel surface and is irrelevant to corrosion) and ***f*** (indicating the light that has reached the steel surface and has been affected by corrosion) propagated leftward across the entire cross-section of the MAIN fiber, crossing the ultrathin adhesive layer into the SUB-1 fiber from Circle-1 and the SUB-2 fiber from Circle-2. The light entering the SUB-1 fiber was disregarded as it could not be monitored. However, the light entering the SUB-2 fiber reached its left end and underwent digital processing together with SUB-2[scattered].

**Figure 7:**
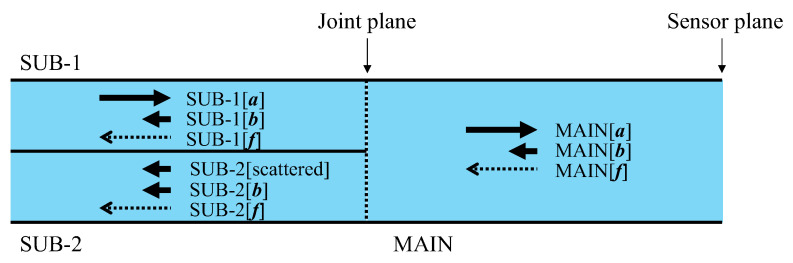
Main, SUB-1, and SUB-2 sections of sensor.

The following summarizes the preceding descriptions of how light travels in each section of the sensor, as illustrated in Figure 7:

1. SUB-1 fiber: A 0.5 mm diameter POF is utilized to transmit light SUB-1 [***a***] from the light source to the MAIN fiber. The SUB-1 fiber receives SUB-1 [***b***] and SUB-1 [***f***] from the MAIN fiber, which is disregarded. Light transmission is bi-directional, but only rightward transmission matters.
2. MAIN fiber: Light MAIN [***a***] received from the SUB-1 fiber is directed to the sensor plane for corrosion observation. A combination of the resulting light MAIN [***b***] (constant light unaffected by corrosion) and MAIN [***f***] (light influenced by corrosion) is forwarded to the SUB-2 fiber. Light transmission is bi-directional, with both rightward and leftward transmission.
3. SUB-2 fiber: Receives the light scattered at the joint plane as SUB-2[scattered] and the light from the MAIN fiber as the measurement result, which is the sum of SUB-2 [***b***] and SUB-2 [***f***]. It transmits them to the image-processing unit. Light transmission is unidirectional.

Thus, the overall trend of the sum of SUB-2[scattered], SUB-2 [***b***], and SUB-2 [***f***] was monitored and analyzed to assess the state of steel corrosion. It was noted that the sum of all light components comprised a constant part *L_c_* (=SUB-2[scattered] + SUB-2[***b***]), which was irrelevant to corrosion, and a varying part *L_v_* (=SUB-2[***f***]), which was influenced by corrosion.

In terms of the sensor structure, the first part (SUB-1 and SUB-2 fibers) comprised two fibers and was designed based on the concept of utilizing light reflection as the fundamental principle of measurement; therefore, it is denoted as R2, where R stands for reflection. As the new sensor has an R2 structure connected to the second part (MAIN fiber), which comprises a single fiber, it is referred to as an R2S sensor.

### 2.3. Manufacturing Method for R2S Sensors

This section outlines the procedure for fabricating the R2S sensors. The sensor was created by bonding 0.5 mm diameter SUB-1 and SUB-2 fibers with a 1 mm diameter MAIN fiber using a readily available instant adhesive (Product ID #04612, KONISHI BOND). The following steps were implemented to ensure the precise positioning of each POF.

**Figure 8:**
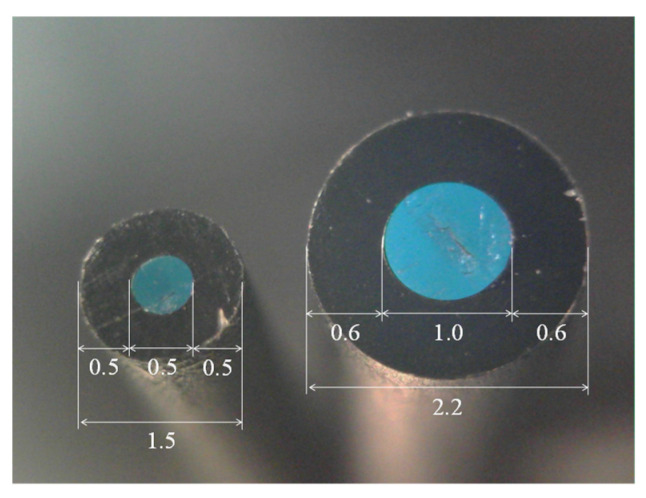
Dimensions of POF cables used to construct R2S sensor (Unit: mm). Note that some blue light was sent into these POFs for photo-taking.

Figure 8 shows a POF cable with a 1.5 mm outer diameter employed for the SUB-1 and SUB-2 fibers (PGS-CD 501-10-E produced by TORAY) and a POF cable with a 2.2 mm outer diameter employed for the MAIN fiber (PGS-CD1001-22-E produced by TORAY). The black regions represent the polyethylene sheaths, while the blue zones indicate the core part of the POF, with diameters of 0.5 mm and 1.0 mm, respectively.

**Figure 9:**
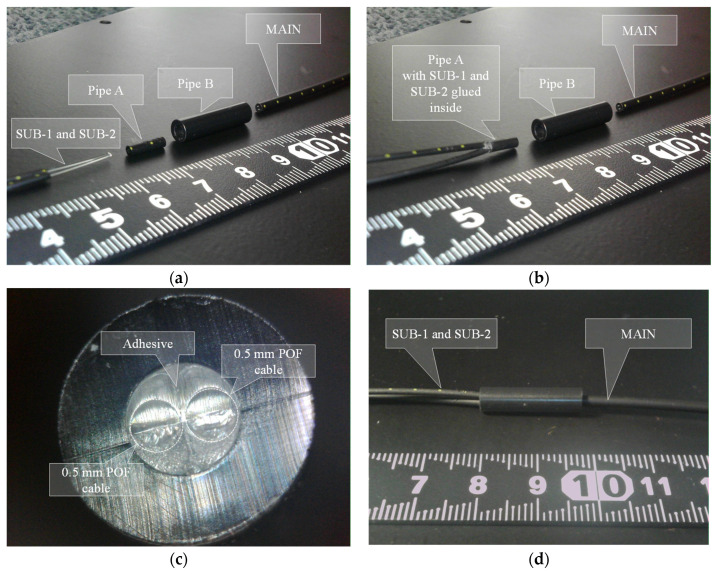
Assembly stages for R2S sensor. (**a**) POF cables and pipes before assemblage, (**b**) after SUB-1 and SUB-2 are set in Pipe A, (**c**) cross-section of Pipe A, and (**d**) completed connection.

To create the R2S sensor, two POF cables with 1.5 mm outer diameters were prepared, and their sheaths were stripped from the ends. Subsequently, a sheath from a POF cable with a 2.2 mm outer diameter was cut to a length of approximately 5 mm. Pipe A (inner diameter: 1.0 mm) was used to hold SUB-1 and SUB-2. Following this, Pipe B (length of 20 mm), with a 2.2 mm inner diameter and a 5.0 mm outer diameter, was prepared. Finally, a POF cable with a 2.2 mm outer diameter was required for the MAIN fiber (refer to Figure 9a). During the preparation of these parts, the left end of Pipe B was modified to have an inner diameter of approximately 3 mm, extending to a depth of approximately 5 mm from the end to securely insert two POF cables with 1.5 mm outer diameters. The basic components are shown in Figure 9a.

**Figure 10:**
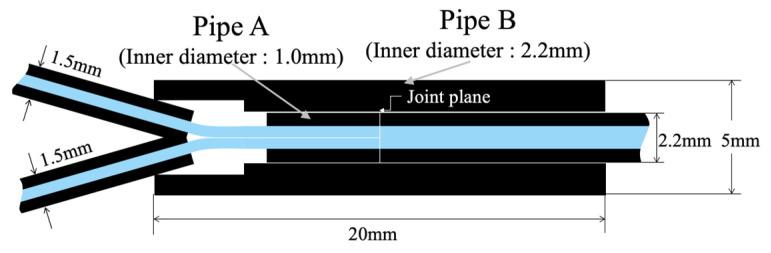
Illustration of longitudinal cross-section of R2S sensor.

Subsequently, as shown in Figure 9b, SUB-1 and SUB-2 were inserted into Pipe A and secured using instant adhesives. The ends were trimmed after the adhesive had adequately dried. Moreover, at this stage, the end of Pipe A took on the appearance depicted in Figure 9c, revealing SUB-1 and SUB-2 (at this point, the distinction between SUB-1 and SUB-2 is inconsequential) within the 1.0 mm inner diameter of Pipe A surrounded by solidified instant adhesive filling the remaining space. The R2S sensor was constructed by bonding the SUB-1 and SUB-2 sides to the MAIN side at the center of Pipe B using an instant adhesive, as illustrated in Figure 9d. The longitudinal cross-section of the connected segment is illustrated in Figure 10. This whole process of manufacturing an R2S sensor could be simplified if a proper coupler specifically designed for this structure were to become available in the future.

## 3. Fundamental Performance of R2S Sensor

### 3.1. Experiments to Validate Functionality of R2S Sensor

**Figure 11:**
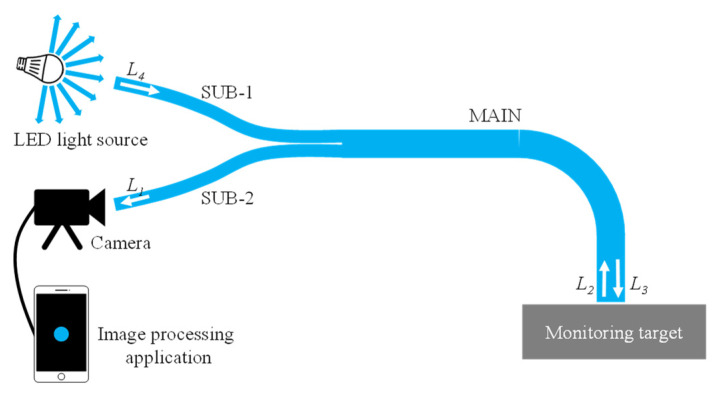
Typical layout of experimental setup for corrosion monitoring by R2S sensor.

Considering the fundamental components explained earlier, the basic layout of the experimental setup for corrosion detection using the proposed R2S sensor is shown in Figure 11. Let us denote *L*_4_ as the initial light intensity when a segment of light emitted from an arbitrary LED source enters the SUB-1 fiber. As this light traverses the SUB-1 fiber, crosses over the joint plane, and reaches the sensor plane through the MAIN fiber, the light intensity diminishes to *L*_3_ (less than *L*_4_) due to the inherent distance attenuation within the polymer material of the POF. Upon reflection from the surface of the object and re-entering the MAIN fiber as *L*_2_, part of this light contains corrosion-related information. After traveling through the MAIN and SUB-2 fibers and crossing over the joint plane again, the light reaches its termination point at the left end of the SUB-2 fiber as *L*_1_. This light, captured by the camera, undergoes image data recognition via an image-processing application on a smartphone.

**Figure 12:**
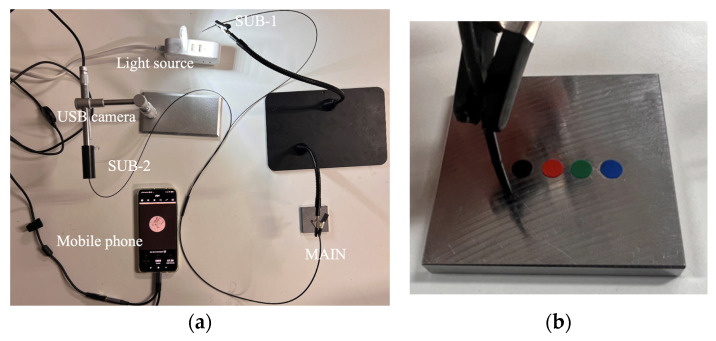
Layout of preliminary experiment. (**a**) Overall layout of preliminary experiment, and (**b**) R2S sensor and steel plate with vinyl tapes of different colors.

A preliminary experiment based on the concept shown in Figure 11 was conducted to validate the functionality of the R2S sensor using the apparatus shown in Figure 12a, where the lengths of the SUB-1, SUB-2, and MAIN fibers were set to 70 cm and 60 cm, respectively.

**Figure 13:**
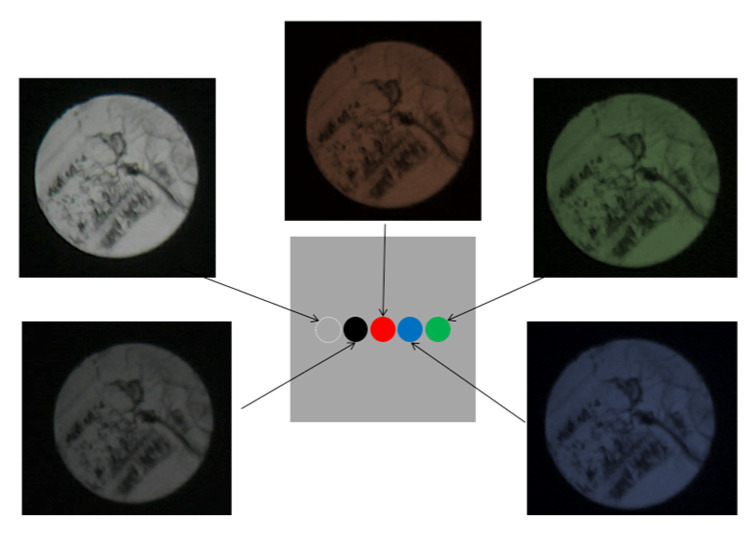
Images of light confirmed by SUB-2 fibers.

As shown in Figure 12b, a steel plate (50 mm × 50 mm × 5 mm) was prepared and adorned with circular vinyl tape of distinct colors (black, red, green, and blue) with a diameter of 5 mm. The sensor plane of the MAIN fiber was placed in direct contact with the steel plate, and its position was changed to the black, red, green, or blue tape spots. The reflected light returning to the end of the SUB-2 fiber at each position was recorded using a microscope camera, as shown in Figure 13. A color change in front of the R2S sensor was identified, thereby confirming its fundamental functionality.

### 3.2. Optical Information Processing Applications

**Figure 14:**
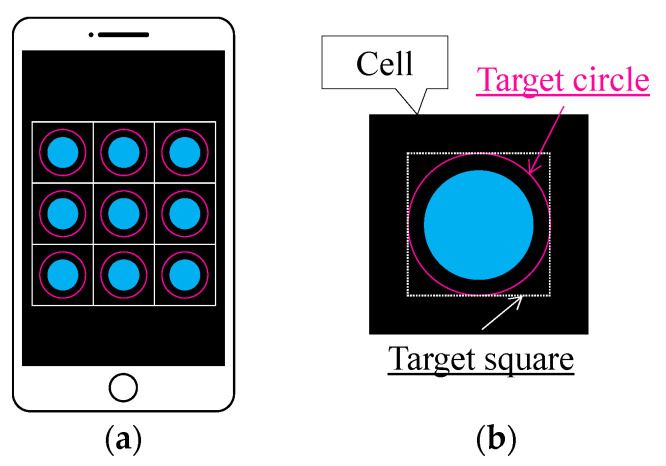
Basic features of graphic application software. (**a**) Multiple POF sensors captured in each cell defined on screen of smartphone (image); and (**b**) definition of target square housing one POF sensor, for which average values of *R*, *G*, and *B* are calculated.

The fundamental capability of the R2S sensor to detect color changes was visually confirmed in a preliminary experiment. However, for broader application in engineering projects, the light detected by the POF sensor must be digitally recorded for more comprehensive tracking of corrosion occurrence and progression. Therefore, a new application software called “image-processing application” [13] that utilizes the image-processing functions of an Android smartphone was employed in this study. The basic features of this system are shown in Figure 14.

In this system, an image of multiple POF sensors arrayed in a regular square grid of cells is first captured by a camera (either the front or backside camera of the smartphone, or by a USB camera attached to the smartphone externally). Subsequently, the positions of the square cells are manually adjusted to capture and encompass all POF sensors in the purple target circles in each cell. Provided that the basic light parameters for any pixel, namely, the red, green, and blue intensities, can be retrieved from the smartphone, one can calculate *R*, *G*, and *B*, which represent the average values of the red, green, and blue intensities for all pixels within the target square, which includes the target circle. The value range for *R*, *G*, and *B* is between 0 and 255. For computational simplicity, a square target square is employed instead of a circular target circle. Therefore, light intensity *L* is a general index that represents the brightness of the measured light:

(1) $$L=\sqrt{R^{2}+G^{2}+B^{2}}$$

This index falls within the range of 0 to 441. The values of *R*, *G*, *B*, and *L* are influenced by the size of the target circle, and as such, they are not regarded as specific physical quantities of light captured by the POF sensor. However, they serve as indices that offer vital insights for tracking deviations from their initial values, thereby enabling the detection and monitoring of corrosion processes.

### 3.3. Simulated Experiment Employing R2S Sensor

**Figure 15:**
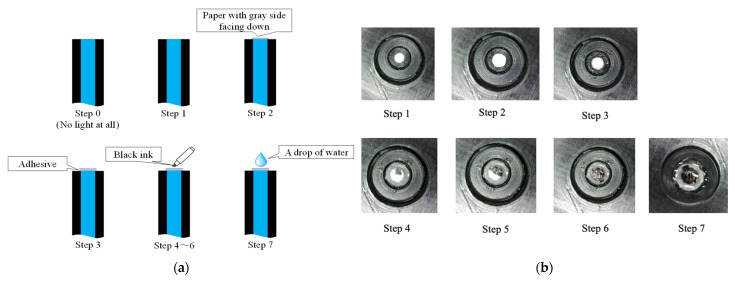
Step of sensing test. (**a**) Sequence of actions taken to check fundamental performance of R2S sensor, and (**b**) photographic images of fundamental experiment.

To validate the functionality of the proposed R2S sensor before conducting an actual corrosion experiment, a preliminary experiment was conducted in a dark room with the R2S sensor oriented upward. The lengths of the SUB-1, SUB-2, and MAIN fibers were set to 70 cm and 60 cm, respectively. Eight sequential steps (steps 0–7), as illustrated in Figure 15, were performed to ascertain proper functioning of the R2S sensor at each stage. In Step 0, with nothing above the R2S sensor, the measurement commenced when the light source was turned off and maintained for approximately 20 s. Subsequently, in Step 1, the light source was activated, directed into the SUB-1 fiber, and held for approximately 20 s. In Step 2, a circular piece of paper with an approximate diameter of 1 mm was cut from regular copier paper, colored gray (to simulate the color of steel), and placed on the R2S sensor with the gray side facing downward. In Step 3, a piece of paper was affixed to the R2S sensor using a commercially available instant adhesive, and its state was maintained for approximately 20 s. In Steps 4, 5, and 6, the top surface of the paper was incrementally marked using an oil-based black marker pen, gradually covering the entire surface with black ink that penetrated the paper strip and was detected using the R2S sensor. In the final step (Step 7), a water droplet was applied to a piece of paper to assess the effect of moisture, and the droplet was held for approximately 2 min.

**Figure 16:**
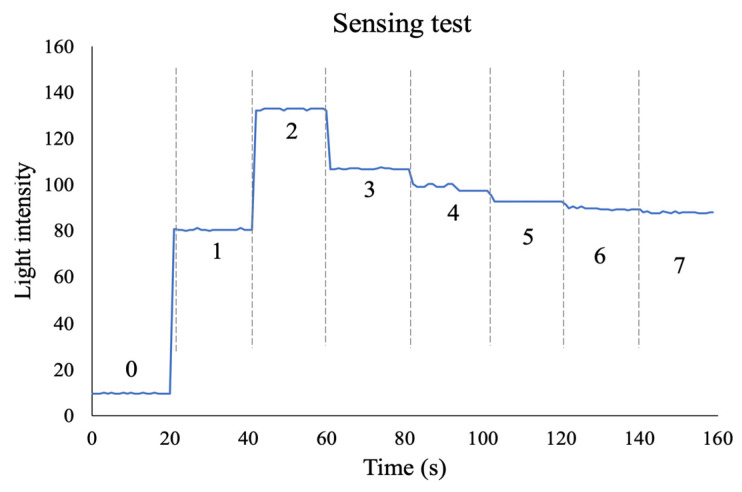
Light intensities recorded in Fibers 1 to 6 during fundamental experiment.

Figure 16 shows the experimental results. In Step 0, the light source was turned off, resulting in nearly zero light being captured by the R2S sensor. In Step 1, the light source was activated, emitting light from the R2S sensor tip into the air. Step 2 involved the placement of a piece of paper on the sensor surface. The light leaving the sensor was reflected through a thin air layer that hit the gray surface of the paper. In Step 3, the paper was glued to the sensor surface by replacing the thin layer of air with an adhesive. The recorded light was darker than that measured in Step 2.

In Steps 4–6, black ink was incrementally applied to the top surface of the paper, directly above the sensor surface, using an oil-based magic marker pen. Consequently, the entire piece of paper gradually became black. Throughout this process, the R2S sensor recorded a gradual decrease in light intensity, mirroring the progressive darkening of the region facing the sensor plane. Finally, to assess the impact of moisture, a water droplet was applied to the paper for permeation. This action marginally decreased the light intensity captured by the R2S sensor, suggesting that, despite a stable color in front of the sensor, a slight fluctuation in the light intensity was observed, which was attributed to moisture.

As demonstrated in this simulation, the proposed R2S sensor can precisely identify alterations in the state of an object surface, primarily focusing on variations in the reflected light intensity. Additionally, it can detect changes in moisture levels.

### 3.4. Maximum Allowable Length of the MAIN Fiber

During measurements with the R2S sensor, there is a concern that the ratio of *L_v_* to the measured light intensity *L* may decrease significantly depending on the length of the MAIN fiber, thereby complicating the interpretation of the measured light intensity. Consequently, a confirmation experiment was conducted to determine the maximum value of *L*_main_ appropriate for measurement purposes.

**Figure 17:**
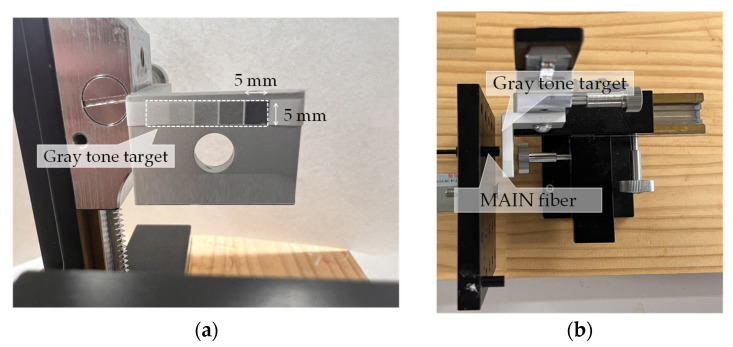
Experimental apparatus. (**a**) Gray tone target paper, and (**b**) overall layout of experimental apparatus.

For this experiment, we prepared a gray tone paper that transitioned from white to black in five increments, as shown in Figure 17. The plane of the R2S sensor was then pressed onto each area (5 mm × 5 mm) to record the measured changes in light intensity. MAIN fiber lengths of 5, 10, 25, 50, and 100 m were utilized. The LED light source and camera employed for image capture were consistent with those utilized in the previous test.

**Figure 18:**
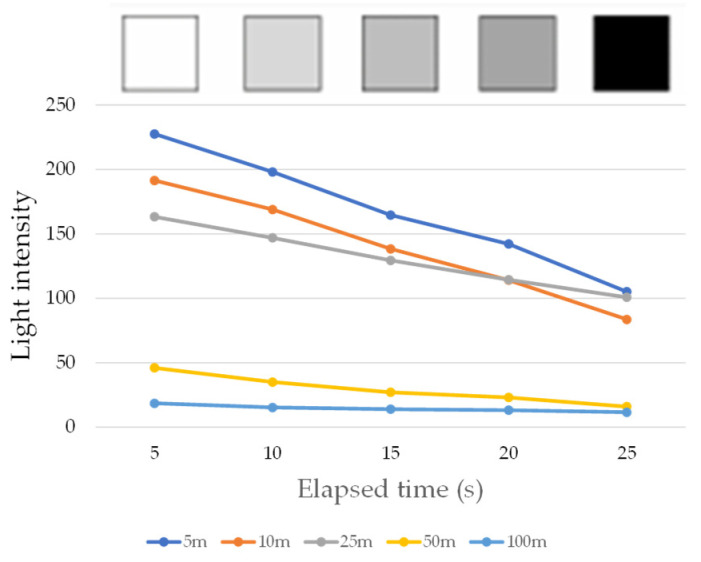
Light intensities recorded during fundamental experiment.

The results of this experiment are depicted in Figure 18. The horizontal axis represents the location of the R2S sensor plane. The decreasing trend of light intensity as the monitored area became darker was noticeable for sensors with *L*_main_ fiber lengths of 5 m, 10 m, and 25 m. However, beyond this point, there was negligible variation in the measured values, suggesting that changes in brightness in front of the sensor plane were not accurately captured. This implies that, with increasing *L*_main_ fiber length values, the proportion of light *L_v_* reflecting the conditions at the tip of the sensor decreased significantly, rendering meaningful measurements unattainable. Consequently, these findings suggest that the *L*_main_ fiber length should be limited to less than 25 m when employing an R2S sensor.

## 4. Corrosion Experiment Using Steel Plate

### 4.1. Basic Strategy for Corrosion Test

A corrosion experiment was conducted to show the performance of the proposed R2S sensor using an SS400 steel plate measuring 50 mm in length, 50 mm in width, and 5 mm in thickness. In the experiment, the bottom side of the plate was exposed to a corrosive environment to track the corrosion phenomenon starting from the bottom and progressing upward until a specified corrosion thickness (*C*) was reached. Moreover, several 2.2 mm diameter holes (with depth denoted as *H*) were arranged on the upper side of the steel plate to facilitate the installation of R2S sensors during the experiment.

**Figure 19:**
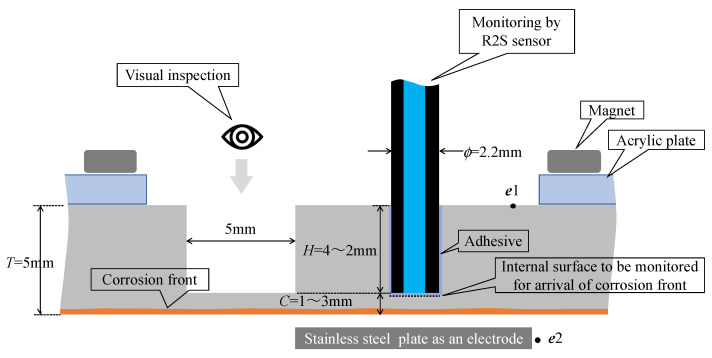
Strategy for corrosion test.

Additionally, observation holes (5 mm in diameter, with a depth denoted as *H*) were formed on the upper side of the plate to observe the arrival of the corrosion zone. Subsequently, points ***e***1 and ***e***2 were connected to the DC circuit for galvanic corrosion testing, as shown in Figure 19. An acrylic fixture was prepared, and neodymium magnets were used to hold the plate at the appropriate height.

### 4.2. Experiment with Three Different Corrosion Thicknesses

**Figure 20:**
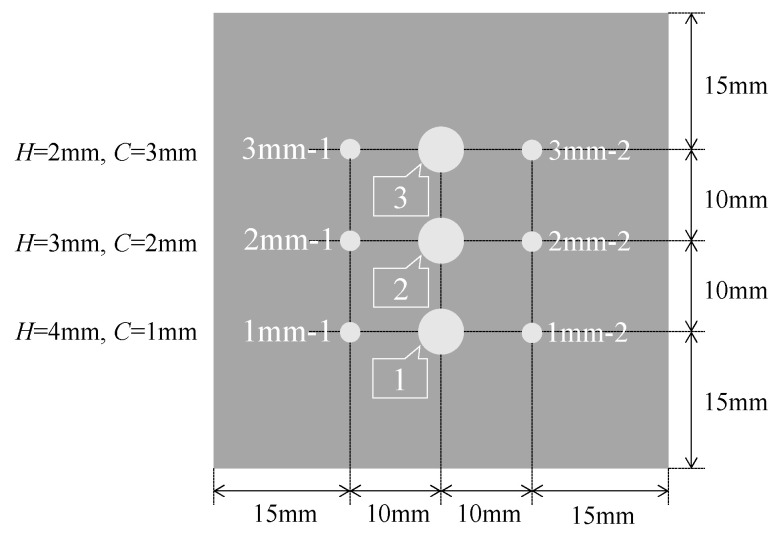
Details of 5 mm thick plate used for corrosion test.

The corrosion experiment aimed to verify whether the progress of the corrosion zone could be systematically captured using the proposed R2S sensors. The corrosion depths (*C*) detected by the R2S sensors were set to 1, 2, and 3 mm. Figure 20 illustrates the positional arrangement of the six sensor holes. For example, the sensor holes (with diameters of 2.2 mm) 1 mm-1 and 1 mm-2 were prepared for *C* = 1 mm. Similar treatments were performed at *C* = 2 mm and 3 mm. Additionally, observation holes 1 (*C* = 1 mm), 2 (*C* = 2 mm), and 3 (*C* = 3 mm), with diameters of 5 mm, were created at the midpoints of the sensor installation holes.

**Figure 21:**
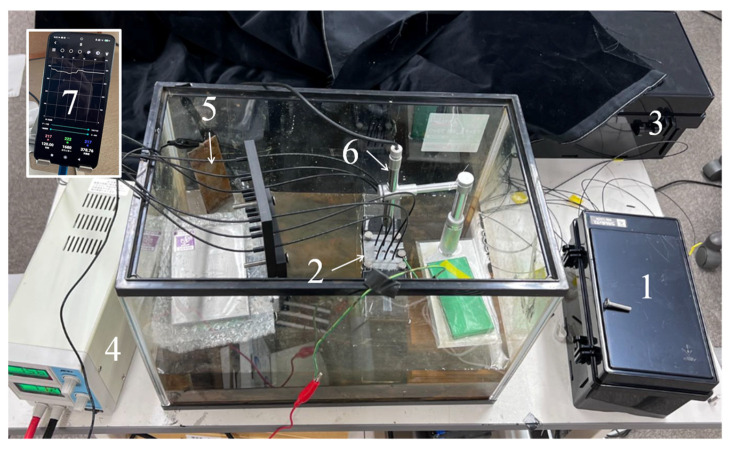
Overall layout of experimental apparatus. (1) Box containing LED light source. (2) Steel plate. (3) Box containing SUB-2 cables from R2S sensors. (4) Direct current control unit. (5) Stainless-steel plate. (6) USB camera used to observe open holes. (7) Mobile phone for graphic image analysis.

Figure 21 illustrates the experimental setup with the steel plate and six R2S sensors installed. A glass container (with a base measuring 600 mm × 300 mm and a height of 400 mm) was filled with a 5% salt brine, reaching approximately half of the steel plate’s height (depicted in (2) in Figure 21). The R2S sensors were affixed to six sensor installation holes on the top surface of the steel plate using Adhesive A, a commercially available instant adhesive. The lengths of SUB-1, SUB-2, and MAIN fibers were set to be 1.5 m and 1 m, respectively.

**Figure 22:**
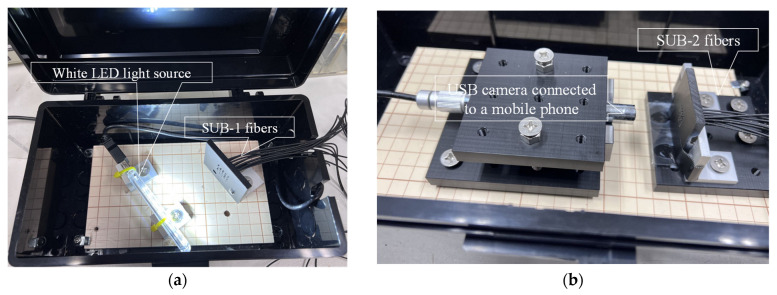
Box design for light source and image recording. (**a**) Typical setup for LED light source box and SUB-1 fibers, and (**b**) typical setup for SUB-2 fibers and USB camera sending visual images to mobile phone.

Light from a white LED light source (1) (refer to Figure 22a for details) was directed to the SUB-1 fiber of each R2S sensor. To record the light captured by each R2S sensor, the SUB-2 fiber was enclosed in a light-blocking box (3) (refer to Figure 22b for details), and images were captured using a USB camera (AS ONE Corporation USB Digital Microscope SDM200) connected to a smartphone (7). In addition, a USB camera (6) was positioned above the observation boreholes and left open to capture images at the bottom of each observation hole. Moreover, device (4) was configured to supply DC for the electric corrosion test, and an electric circuit was established by connecting the electrodes from this device to the steel plate and a stainless-steel plate submerged in the brine (5).

## 5. Experimental Results

Subsequently, electrolytic corrosion tests were conducted at an initial voltage of 30 V. The corrosion test lasted for 50 h after confirming sufficient corrosion conditions at observation hole 3, where the corrosion depth was set to 3 mm.

**Figure 23:**
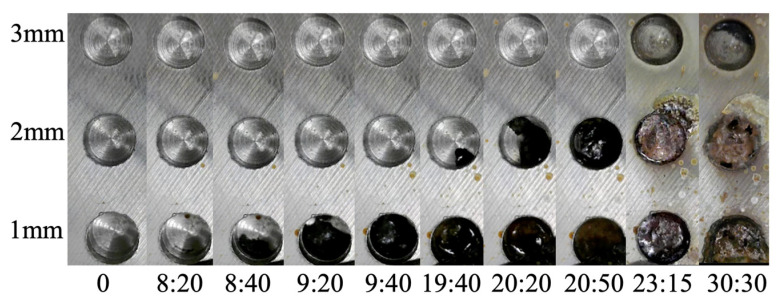
Images recorded at observation holes, showing progress of corrosion.

Figure 23 shows the recorded conditions at several experimental stages in observation holes 1–3, designated for visual observation over the approximately 50 h experiment duration. In each image, the lower row corresponds to observation hole 1 (*C* = 1 mm), the middle row to observation hole 2 (*C* = 2 mm), and the upper row to observation hole 3 (*C* = 3 mm). The “hour: minute” notation below each photo indicates the elapsed time from the start of the experiment.

At approximately 8:25 (8 h and 25 min into the experiment), a black area emerged at the base of observation hole 1, indicating that a portion of the corrosion front originating from the underside of the steel plate reached a corrosion thickness of 1 mm. By 9:40, the corrosion area had expanded, covering the entire area of borehole 1. Approximately 10 h into the experiment, the corrosion products became visible on the lower side of observation hole 2 at approximately 19:30. By 20:50, the entire bottom of the hole was coated with corrosion products. In observation hole 3, the corrosion products emerged on the upper-right side of the bottom of the hole at 30:15, and the entire area was covered at 31:40.

In summary, the corrosion zone advanced by approximately 1 mm in 10 h, and it took approximately 1 h from the initial appearance of the corrosion products at the bottom of each observation hole to cover the entire area.

**Figure 24:**
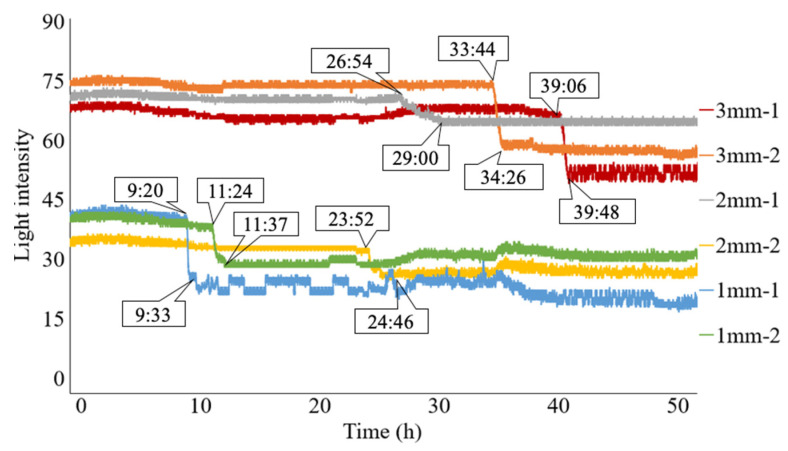
Light intensities recorded for all R2S sensors.

Figure 24 shows the light intensities recorded during the occurrence and progression of the corrosion phenomena captured by the six R2S sensors throughout the 50 h experiment. The timestamps shown in the figure represent **t**_i_ (the time at which the light intensity started to decrease) and **t**_f_ (the time at which the decreasing behavior of the light intensity roughly stopped) for each R2S sensor.

During the first 9 h of the experiment, there were no notable data fluctuations in any of the R2S sensors, with each sensor maintaining a stable light intensity level. The differences in the recorded light intensities among the sensors during this period resulted from subtle variations in the sensor characteristics due to manual manufacturing procedures. However, the initiation and progress of the corrosion can be discerned by observing the deviations from the respective initial values.

**Figure 25:**
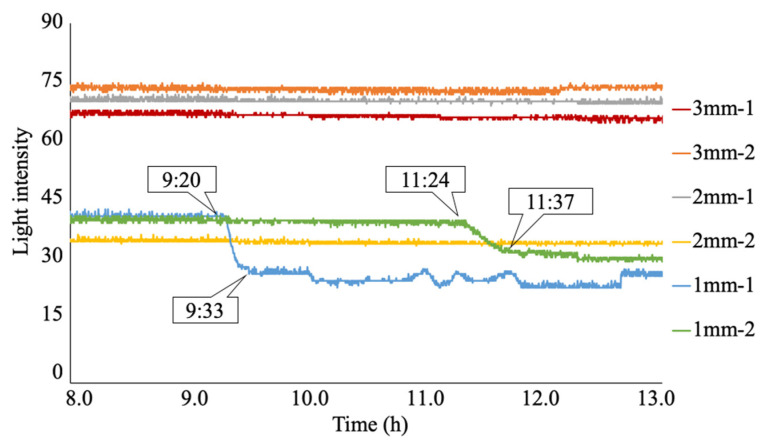
Expanded view of light intensities recorded for 1 mm-1 and 1 mm-2.

The R2S sensor installed at 1 mm-1 was the first to detect the arrival of the corrosion zone. To examine the behavior of the light intensities when the corrosion front reached the bottom of observation hole 1 mm-1 in more detail, we expanded the elapsed time range from 8:00 to 13:00, as shown in Figure 25. At approximately 9:20, the light intensity started to decrease due to corrosion. This behavior continued and ceased at approximately 9:33, when the entire area in front of the sensor plane was corroded. Thus, the progressive nature of the corrosion zone was captured from the initial arrival of the corrosion zone, even in the small area monitored by the R2S sensor.

Subsequently, the light intensity for the sensor at 1 mm-1 remained in a somewhat unstable state of data fluctuation until the experiment’s conclusion. This instability persisted until the final stage of the experiment because of its tendency to be influenced by the corrosion products (a mixture of black, brown, and various colors, including water) generated during the ongoing corrosion process.

The second arrival of the corrosion zone was detected by the sensor installed at 1 mm-2 at 11:24. Subsequently, the light intensity of the sensor declined sharply until approximately 11:37, when the bottom of the hole was completely covered by the corrosion zone. Subsequently, it exhibited stable behavior until the final stage, with occasional fluctuations. Despite some subsequent changes, the sensor maintained regular behavior until the end of the experiment.

For the sensors installed at 2 mm-1 and 2 mm-2, the arrival of the corrosion zone was recorded at 26:54 and 23:52, respectively. The subsequent variation in light intensity exhibited a similar pattern to those observed at 1 mm-1 and 1 mm-2.

Finally, for the sensors installed at 3 mm-1 and 3 mm-2, the arrival time of the corrosion zone was recorded at 39:06 and 33:44, respectively, indicating that the corrosion thickness reflected the difference in the arrival time of the corrosion zone.

Table 1 summarizes the timeline of the onset and development of corrosion events recorded by each sensor. Here, Δ***t*** represents the time interval (***t****_f_*-***t****_i_*) for the corrosion zone to initially reach the observed area of the sensor and cover the entire monitored region. The time interval varied between 0:13 and 2:06.

**Figure 26:**
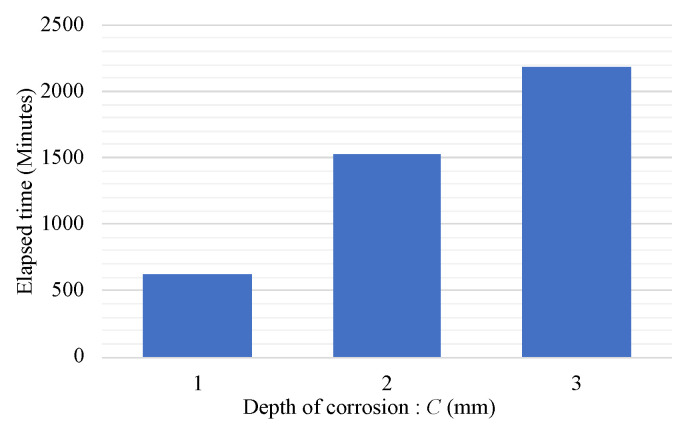
Relationship between averaged time of first corrosion zone arrival and corrosion thickness.

Additionally, because two R2S sensors were employed for each corrosion depth, the average time for the initial capture of the corroded zone is shown in Figure 26. From these results, it is evident that by installing the proposed R2S sensors at different depths, the progression of corrosion areas can be quantitatively and clearly understood.

**Figure 27:**
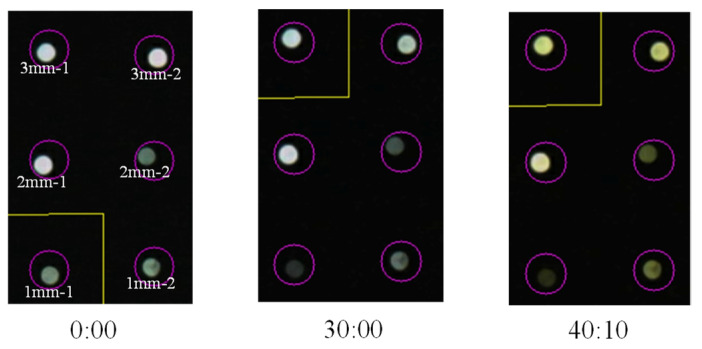
Screenshot images of light observed for R2S sensors during test.

The analysis relies on the light intensity captured by the R2S sensor. However, the image-processing application used in this study recorded screenshots depicting the state of the POF during monitoring. Figure 27 shows screenshots captured at elapsed times of 0, 30:00, and 40:10. As is evident from these records, given that the R2S sensor orchestrated the detection of the arrival of the corrosion zone, it is highly feasible to identify the initiation and progression of corrosion phenomena by examining the alterations in light intensity and color with the naked eye.

## 6. Experiment Using Small Dummy Sensor

### 6.1. Necessity of Small Dummy Sensors

Based on the experimental results described above using a steel plate, it was demonstrated that the R2S sensor could accurately detect the propagation of corrosion zones. However, in real-world structural monitoring scenarios, challenges may arise where the location and direction of the corrosion zone are unknown, or it may be excessively difficult to drill into the existing structure to install the R2S sensor. In such situations, employing a dummy sensor to monitor corrosion and its progression would become a prudent approach for indirectly estimating the state of corrosion on an actual structure within a similar environment. To achieve this objective, a compact dummy sensor was constructed, and its functionality was validated using insights obtained from the plate corrosion experiment.

### 6.2. Fundamental Configuration of Compact Dummy Sensors

**Figure 28:**
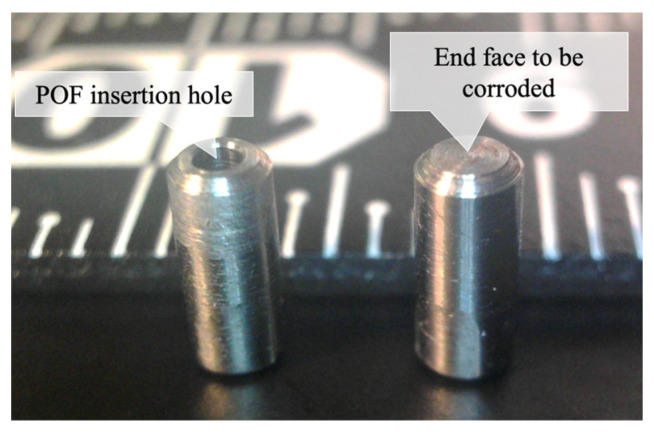
Crafted steel rods used for dummy sensor.

**Figure 29:**
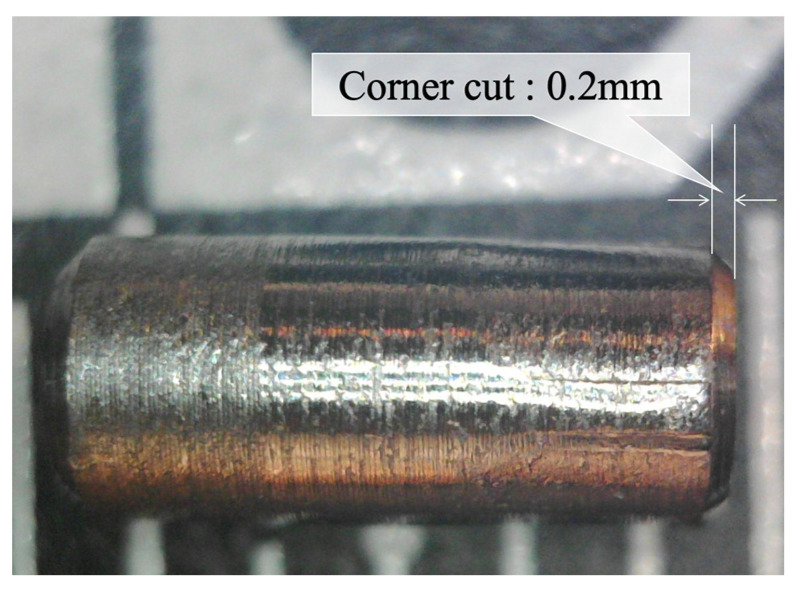
Side view of dummy specimen.

To create a compact dummy sensor, a steel rod with an outer diameter of 2.2 mm and a length of 5 mm was crafted. The rod was drilled from one side with a 1.0 mm diameter drill, leaving a corrosion thickness *C* on the opposite side. Figure 28 depicts two pieces of crafted steel rod, with one on the left displaying the POF insertion hole facing upward and the other on the right showing the face to be corroded pointing upward. The ruler in the background provides a scale of 1 mm per division. Figure 29 shows a side view of the dummy specimen.

**Figure 30:**
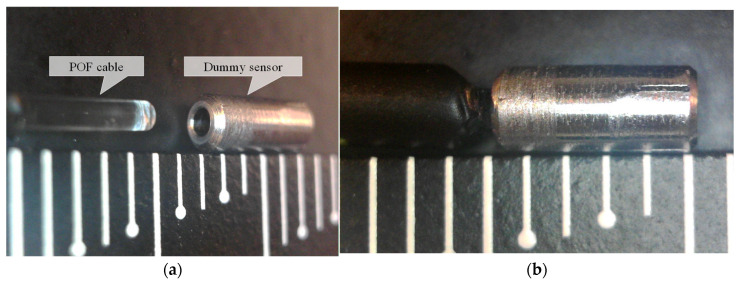
Assembly steps for dummy sensor. (**a**) POF before insertion into dummy sensor, and (**b**) dummy sensor with POF already inserted.

To complete the construction of the compact dummy sensor, the tip of the MAIN fiber was uncovered to expose a POF approximately 4–5 mm in length (Figure 30a). Subsequently, it was inserted and securely fixed into the dummy sensor using a small amount of instant adhesive (Figure 30b).

### 6.3. Strategy for Corrosion Experiment

**Figure 31:**
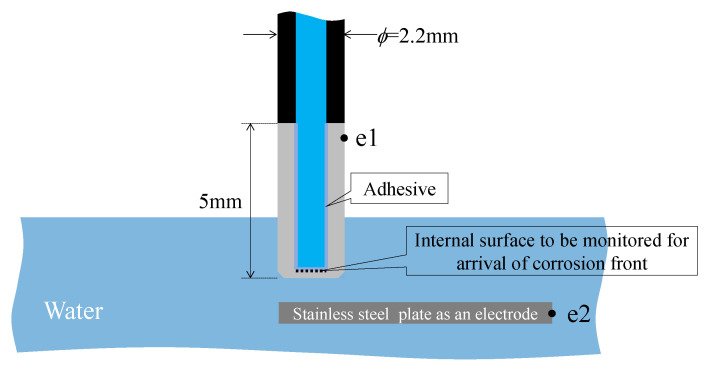
Fundamental strategy for corrosion test.

**Figure 32:**
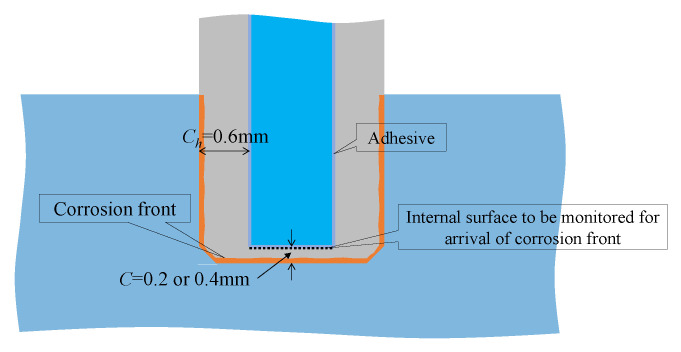
Magnified image of immersed area.

Figure 31 depicts the corrosion experiment conducted using the dummy sensor. The 5 mm long tip of the dummy sensor was submerged in water, establishing an electrical circuit by connecting an electric wire to point ***e***1 on the top of the dummy sensor. Another electrode, point ***e***2, was positioned inside the tank containing 5% salt brine. In this experiment, two dummy sensors with 0.2 mm and 0.4 mm corrosion thicknesses were corroded simultaneously, necessitating the installation of two electric circuits for each sensor. A magnified image of the immersed area is presented in Figure 32.

The dummy sensor’s body featured a wall thickness (denoted as *C_h_* in the figure) of 0.6 mm. Additionally, a segment with a corrosion thickness designated as *C* was positioned at the bottom. Under these conditions, it is assumed that corrosion initiated from all immersed parts at the start of the electric corrosion experiment and that the corrosion zone propagated uniformly. Consequently, with a corrosion thickness *C* of either 0.2 mm or 0.4 mm at the bottom, which is smaller than *C_h_* (0.6 mm), any alteration in the optical data recorded by the R2S sensor would indicate that the corrosion area, originating and spreading from the bottom, achieved a corrosion thickness of *C*.

### 6.4. Corrosion Experiments and Results

The experiment consisted of two trials (Cases 1 and 2) aimed at validating reproducibility. In each case, dummy sensors with 0.2 mm and 0.4 mm corrosion thicknesses were employed. The length of the MAIN fiber, denoted as *L*_main_, was fixed at 2 m for both cases. The names of the dummy sensors used in each case are detailed in Table 2.

**Figure 33:**
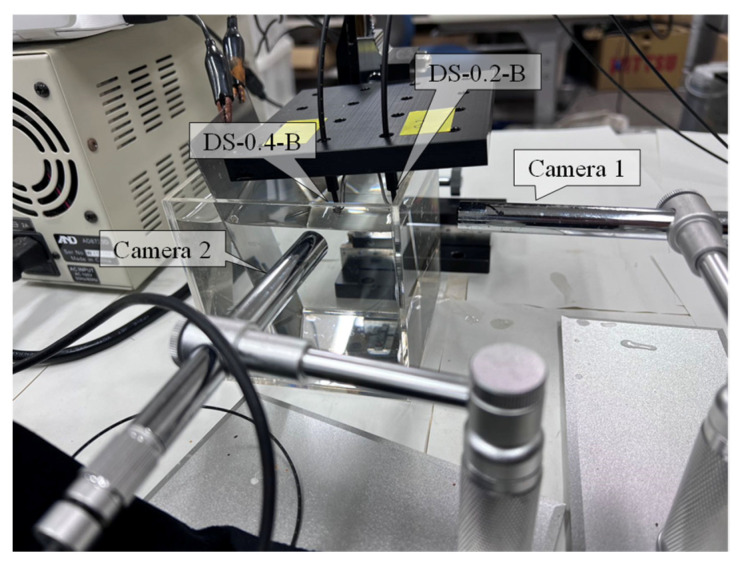
Overall layout of dummy corrosion test.

Due to the compact size of the dummy sensors utilized in this experiment, the entire apparatus was arranged within a relatively small acrylic container measuring 100 mm in width, depth, and height. Two USB cameras, labeled Camera 1 and Camera 2, were positioned externally near the container, as depicted in Figure 33, to observe the corrosion process of the dummy sensors.

**Figure 34:**
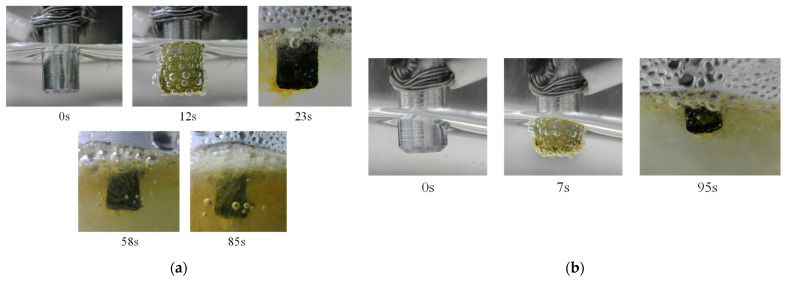
Images recorded by cameras. (**a**) DS-0.2-B recorded by Camera 1, and (**b**) DS-0.4-B recorded by Camera 2.

Given the diminutive dimensions of the dummy sensor, the corrosion experiment typically concluded within a maximum duration of approximately 3 min. Throughout this period, bubbles and yellowish/brownish liquid emerged on the surface of the dummy sensors as the electrolytic corrosion process progressed, as shown in Figure 34 for the experiment conducted in Case 2 (only one camera was used in Case 1).

**Figure 35:**
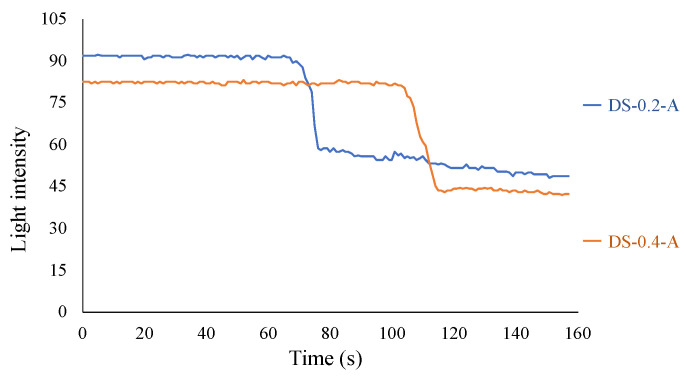
Light intensities recorded for R2S sensor installed in DS-A.

The recorded light intensities for Case 1 are shown in Figure 35. In DS-0.2-A (dummy sensor with a corrosion thickness of 0.2 mm), the decline in light intensity commenced at 68 s. It took approximately 10 s to reach the point at which the decrease was temporarily halted. For DS-0.4-A (dummy sensor with a corrosion thickness of 0.4 mm), the reduction in light intensity initiated at 105 s, lasted for approximately 10 s, and then stabilized. The time needed to reach the corrosion zone was not strictly proportional to the corrosion thickness, but generally mirrored this distinction.

**Figure 36:**
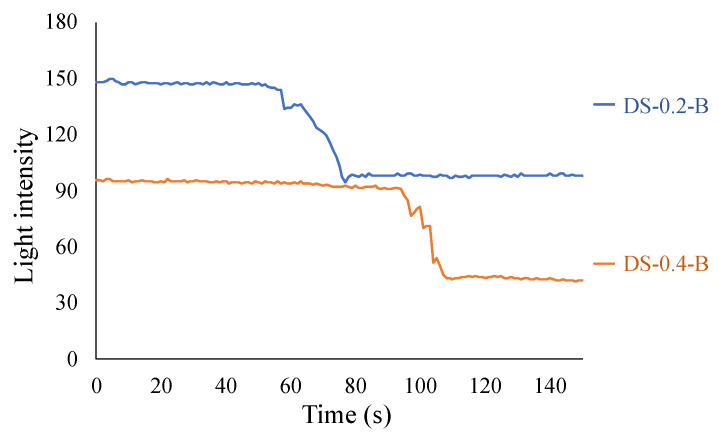
Light intensities recorded for R2S sensor installed in DS-B.

The results of Case 2 are depicted in Figure 36. In this instance, the light intensity of DS-0.2-B began to decrease at approximately 58 s and persisted for approximately 18 s before stabilizing. For DS-0.4-B, the reduction in light intensity initiated at 95 s, lasted for approximately 15 s, and then stabilized. These patterns mirror those observed in Case 1, thus substantiating the reproducibility of the corrosion experiments.

**Figure 37:**
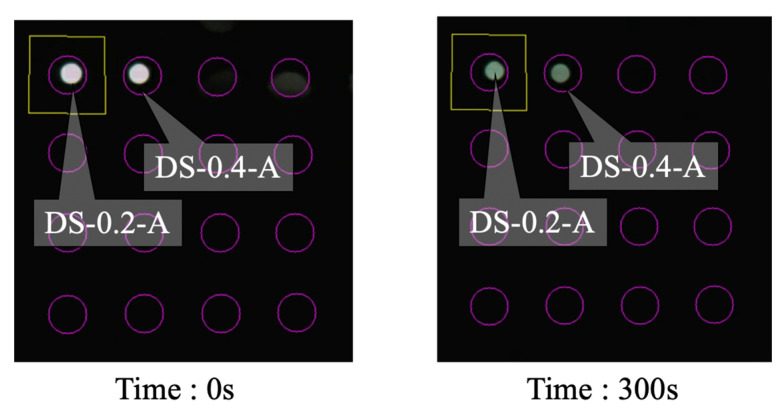
Screenshot images of light observed for R2S sensors during Case 1.

**Figure 38:**
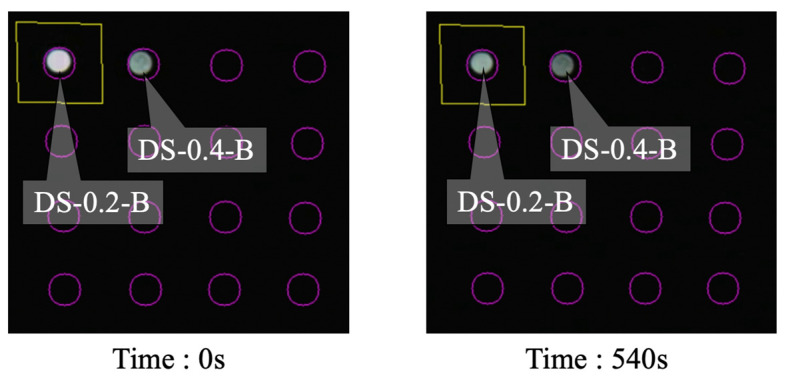
Screenshot images of light observed for R7 sensors during Case 2.

Screenshots capturing the initiation and conclusion of the experiment are presented in Figure 37 and Figure 38 for Cases 1 and 2, respectively. As is evident from these screenshots, the arrival of the corrosion zone can be adequately verified with the naked eye.

## 7. Conclusions

This paper introduced a novel plastic optical fiber sensor designed using the concept of bi-directional light transmission to monitor the progress of corrosion in steel plates. The expected functionalities of the proposed sensor were validated through several experiments, leading to the following conclusions:

1. A new POF sensor that optimally employs the concept of bi-directional light transmission was proposed and demonstrated to be suitable for corrosion detection.
2. Owing to its structure, which comprises two POF cables (SUB-1 and SUB-2 fibers: diameter *D*_2_, length *L*_sub_) and another POF cable (MAIN fiber: diameter *D*_1_, length *L*_main_), a sensor can be produced in which the majority is made of MAIN fibers. This design leads to a significant reduction in the overall number of POF cables required to monitor corrosion at multiple points.
3. The sensor installation procedure has been improved to be extremely simple, such that the only preparation required is to puncture a sensor installation hole (2.2 mm in diameter in this case) of the desired depth before securely affixing the sensor into the hole using an instant adhesive.
4. It is advisable to limit the length of the MAIN fiber to 25 m or less when utilizing the R2S sensor to ensure meaningful outcomes.
5. An analysis of the data collected by various R2S sensors installed at different corrosion depths revealed that the incremental corrosion progression of the steel plate could be qualitatively captured in the corrosive environment of this experiment.
6. The progress of the corroded area within a small zone monitored in front of the sensor plane was identified. The average value of the time interval Δ***t*** was 48 min under corrosive conditions in the steel plate experiment.
7. The progression of the corroded zones identified using the R2S sensors was adequately identified by the naked eye.
8. An extremely compact dummy sensor attached to the R2S sensor tip, with a diameter of 2.2 mm and a length of 5 mm, effectively recorded the complete corrosion phenomenon at the designated corrosion thickness *C* by ensuring that the thickness of the tip was smaller than that of the body segment *C_h_*.

As stated in the conclusion, using the R2S sensor proposed here ultimately minimizes the amount of POF cable needed. However, it is important to note that there is a limit to the sensor’s effectiveness in acquiring data; its length is required to be kept below 25 m. Additionally, since individual differences in sensors may arise during the connection of two thin POFs to the MAIN cable, future manufacturing of components such as couplers to ensure consistency in the quality of these connections will be necessary. Furthermore, the compact dummy sensor presented lastly is exceptionally small, enabling its use in extremely confined spaces where conventional corrosion sensors cannot be installed, thus holding promise for diverse applications across various fields.

## Figures and Tables

**Table 1:** Recorded time of events (hour:minute).

Sensor Location	Elapsed Time at Which Corrosion Was Detected for the First Time: *t_i_*	Elapsed Time at Which the Reduction in Light Intensities Was Stabilized: *t_f_*	Time Required for Reduction in Light Intensities: Δ*t* = *t_f_* − *t_i_*
1 mm-1	9:20	9:33	0:13
1 mm-2	11:24	11:37	0:13
2 mm-1	26:54	29:00	2:06
2 mm-2	23:52	24:46	0:54
3 mm-1	39:06	39:48	0:42
3 mm-2	33:44	34:26	0:42
-	-	-	0:48 (averaged)

**Table 2:** Experiment cases and names of dummy sensors.

Case	*C* = 0.2 mm	*C* = 0.4 mm
1	DS-0.2-A	DS-0.4-A
2	DS-0.2-B	DS-0.4-B

## Data Availability

Data are contained within the article.
